# Preuniversity Students' Perceptions and Attitudes About an Anatomy and Physiology Outreach Program: Survey Study and Inductive Thematic Analysis

**DOI:** 10.2196/52533

**Published:** 2024-08-12

**Authors:** Ranganath Vallabhajosyula, Vivek Perumal, Ramya Chandrasekaran, Sreenivasulu Reddy Mogali

**Affiliations:** 1 Department of Anatomy Lee Kong Chian School of Medicine Nanyang Technological University Singapore Singapore

**Keywords:** anatomy, physiology, educational outreach, preuniversity outreach, team-based learning, anatomy workshop, medical education, health profession education, health professions, health care careers, student, students, outreach activity, physiological, school, university, Singapore, thematic analysis, learning, education, motivation, health care, health profession, medical school

## Abstract

**Background:**

Science and health outreach activities are aimed at motivating and sparking interest among prospective students to pursue careers in these fields; however, research studies supporting this hypothesis are limited.

**Objective:**

The aim of our study was to organize an integrated Anatomy and Physiology outreach to examine preuniversity students’ learning experiences (learning tools, activities, and facilitators) and motivation to pursue a career in health care and to gather evidence on their attitudes and perceptions of such activities.

**Methods:**

A 2-day course on cardiorespiratory and gastrointestinal anatomical systems was presented at the Lee Kong Chian School of Medicine in Singapore using its key pedagogies, that is, multimodal practical and team-based learning. Ninety preuniversity students from 21 preuniversity institutions in Singapore participated in this 2-day course, and their experiences were evaluated using a 4-point Likert scale and open-ended survey questions. Free-text comments were analyzed using inductive thematic analysis.

**Results:**

The survey using the 4-point Likert scale was completed by 81 (92%) of the 88 participants. Most students felt that the course materials were adequate (mean 3.57, SD 0.57) and met the learning objectives (mean 3.73, SD 0.52). The students felt that the instructors were clear (mean 3.73, SD 0.52) and effective (mean 3.70, SD 0.53). They liked the organization of the outreach session (mean 3.64, SD 0.48) and were highly motivated to study medicine or allied/biomedical sciences (mean 3.69, SD 0.54). Practical and team-based learning were regarded as exceedingly satisfactory (mean 3.63, SD 0.53 and mean 3.58, SD 0.54, respectively). All the respondents said that they would recommend this course to peers. Thematic analysis revealed that the participants gained a new perspective of the human body structure and function, they liked the unique learning settings, they were motivated to pursue a career in health care, they were satisfied with the sessions, and interactions with the facilitators increased their understanding of the human anatomy and physiology.

**Conclusions:**

Structured health outreach activities provide students with unique opportunities to experience a preclinical learning environment in a medical school, deepen their understanding of human body structure and function, and increase their motivation and interest in science. Further, outreach programs may lay the foundations for potential students aiming to pursue health profession education.

## Introduction

### Background

The majority of preuniversity students position education at the forefront of their aspirations for a successful future [1]. They explore opportunities that multiply their individual interests in science, technology, engineering, and mathematics (STEM) education, in the form of outreach programs by universities, which provide a wide range of engaging activities to involve and inspire the next generation. Such initiatives introduce students to higher education, bridge the gap between classroom knowledge and real-world experience, and ultimately build their confidence in pursuing STEM education [2].

Typically, outreach activities in STEM education are developed and executed by experts to provide a unique learning experience for younger generations outside the classroom [3]. The nature of outreach programs ranges from introductory to developmental to consolidation based on student learning experiences [4-6]. The commonly employed activities in outreach are roadshows, guest lectures, campus tours, workshops, demonstrations, and summer schools [4-11]. These events give an opportunity for widening participation in raising goals and encouraging underrepresented students (from lower socioeconomic and lower income groups) to enter higher education [12,13]. The most valuable outcomes from the events are not only the opportunities for education, engagement, or the development of interest but also inspiration from senior students and faculty of the participating institutions [14]. This is vital in supporting young people in their decision-making and suitability for a future career, for example, in medicine [15].

Existing studies have shown that undergraduate anatomy and physiology students who were exposed to anatomy observational outreach through multimodal teaching techniques showed enhanced knowledge and interest in STEM and allied health degrees [8,9]. There is also evidence that even shorter and flexible programs and extra academic discussions such as body donation increased students’ understanding of human anatomy [16]. These studies [4-11,16] demonstrate the significance of outreach programs in fostering students’ engagement and motivating them toward a career-driven program. However, in the current literature, a detailed outreach program that is integrated with pedagogical practices is limited [9]. We propose that when anatomy and physiology outreach is combined with student-centered pedagogy, it would deepen students’ understanding of the human body and ignite students’ interest and motivation to make an informed decision about their future career.

### Theoretical Framework of This Study

The main objective of the outreach activity in our study was to provide a unique learning opportunity for preuniversity students. This involved acquainting them with the preclinical learning environment of a medical school and fostering their enthusiasm for pursuing a career in medicine, health care, and biomedical sciences. The self-determination theory [17] provides the theoretical framework for understanding the motivation in this context.

Engaging learning materials and student-peer-faculty collaboration contribute to a stimulating learning environment in which students can apply prior knowledge in real-world contexts and discover personal relevance [17,18]. Learning satisfaction is the fulfilment that learners experience when their needs and requirements are met through learning activities [19]. This satisfaction is influenced by factors such as course content, teaching modalities, learning convenience, and interactions with peers and instructors [20]. The self-directed learning approach cultivates external motivation in individuals by influencing rewards or grades and internal motivation by influencing innate satisfaction and personal interests [17], which in turn have been shown to improve student attitude toward STEM courses and career choices [21]. By addressing these fundamental requirements and developing a structured and engaging learning activity, an outreach program can increase interest in the topic and can be applied for increasing interest in other professions in the future [21].

### Context of This Study

Although there are studies in the literature that have used outreach programs in medical schools to spark interest among preprimary-to-high-school students [8,10], there is sparse information in the Asian context on the effectiveness of structured outreach programs that mimic actual preclinical integrated anatomy and physiology learning experiences for preuniversity students. One Asian study by Simok et al [22] described an outreach program in conjunction with the World Anatomy Day. It was essentially an engagement session for visitors, including students, faculty, and staff across various departments and universities. The event comprised activities such as puzzle games, quizzes, and other fun-related activities related to anatomy. Another study [23] reported a program that offered a tour of various departments; these activities could have been informative on the organization’s facilities, infrastructure, and programs offered rather than providing structured learning opportunities for the prospective university students to explore the organization’s pedagogy and educational resources.

To stand out from the typical outreach programs that are basically designed as open days and awareness events, the Lee Kong Chian School of Medicine (LKCMedicine) conducted a 2-day outreach program (workshop) for preuniversity students in Singapore; this event was titled as “Heart for Medicine and Guts for Medicine.” LKCMedicine is the most recently established medical school in Singapore, offering a 5-year Bachelor of Medicine and Bachelor of Surgery (MBBS) degree. LKCMedicine has a system-based integrated curriculum in the preclinical years (first 2 years), delivered via team-based learning (TBL) pedagogy with appropriate anatomy and science laboratories. The major focus of this event was to introduce preuniversity students to the actual medical school learning environment (pedagogy, educational tools, and infrastructure), guided by medical students and faculty.

We understand that a successful learning environment should have 5 essential features, namely, context, selection, location, objective, and a teaching method that shapes the perceptions and prospectus of participants as well as nurtures their motivation and learning [24,25]. Our outreach program meets these criteria: being conducted in an educational context by carefully selecting preuniversity students, utilizing a location where medical students routinely study, employing an objective, and using a teaching method aligned with the medical curriculum (TBL and hands-on practical) and the program’s goal. In addition to the main objective of the outreach activity, that is, providing a unique learning experience, this study aims to investigate the perceptions of the participants over the 2-day structured outreach program through a questionnaire survey. This study analyzes and reports the students’ learning experiences and the utility of a 2-day outreach program based on the survey data and the open-text comments of the participants.

## Methods

### Workshop Theme and Process

Given that potential medical graduates need a whole “heart” (dedication) for medicine and “guts” (courage) to practice medicine, an event theme based on the anatomy of the cardiorespiratory and gastrointestinal system was chosen. The activities in the program were similar to the learning environment for anatomy practical and TBL-based anatomy and physiology instruction in LKCMedicine. The learning outcomes of the session are presented in Multimedia Appendix 1.

### Participant Recruitment

The outreach invitation (a 2-day anatomy and physiology workshop) was extended to all public polytechnic and junior colleges in Singapore. To ensure fair representation from each school, we applied a limit of 5 students per institution. The nomination of students was left to the discretion of each school. However, certain criteria such as academic achievement, scientific interest, and collaboration or leadership qualities were specified. It was strongly recommended that 1 of the 5 pupils from each institution receive financial assistance for their studies; this was consistent with the percentage of LKCMedicine medical students receiving financial support.

### Outline of the Educational Outreach Session

#### Teaching Topics

Cardiorespiratory and gastrointestinal systems were chosen for this session to align with the theme of outreach. The main rationale to choose these topics was that both cardiorespiratory and gastrointestinal systems are usually considered easier than other body systems, for example, the nervous system [26]. Another reason for this consideration was that these systems are usually taught in high schools at least at an introductory level. Further, the structures comprising these systems are usually large and distinguished enough for easy visualization and demonstration. On the contrary, the musculoskeletal system has complex details to visualize and remember because of similar structures such as tendons, vessels, and nerves. This rationale is further supported by evidence in the literature on the perceived difficulty of the organ systems [26]. These factors were carefully considered by the authors while developing the themes for the outreach program.

#### Duration and Facilitators of the Outreach Session

The basic anatomy and physiology of the cardiorespiratory and gastrointestinal systems were covered over the course of 2 days (6 hours per day). The teaching and learning were managed by a multidisciplinary team of content experts (anatomy, radiology, surgery, and physiology). In addition, LKCMedicine MBBS students also assisted in facilitating the practical and TBL activities.

#### Learning Materials

The course consisted of hands-on anatomy practical sessions and TBL sessions. The practical was performed in the morning, and TBL was delivered in the afternoon on the same day. To support practical teaching and learning, we used plastinated human bodies and organs, Anatomage (3D virtual dissections), plastic models, and imaging. In addition, a practical handout highlighting the learning objectives and structures that need to be identified in the specimens was provided. For the TBL sessions, PowerPoint slides were prepared for the students and given 2 weeks prior to the outreach program so that they could prepare for the session. The TBL questions such as the individual readiness assurance test, team readiness assurance test, and application exercises were made by content experts. All these activities were in accordance with the routine teaching style followed at LKCMedicine.

#### Teaching and Learning Activities

Day 1 of the outreach program focused on cardiorespiratory anatomy and physiology, and day 2 focused on the gastrointestinal system. For student diversity, they were sorted into 5-member teams based on their school, level of study, and sex; no more than 2 students from the same school were placed in any team. The same team was used for both day sessions. A brief icebreaker session was conducted at the beginning so that the team members could get to know each other better.

During the practical (morning session of about 3 hours), the students were divided into 3 groups and were assigned to 1 of the 3 learning stations (2 gross anatomy and 1 imaging station). In the practical sessions, the students were asked to identify the organs and their associated bones, blood vessels, nerves, and their topographic arrangement and key anatomical relationships by using the multimodal practical resources and discuss the features within the teams as well as with the facilitators. These practical sessions align with the learning outcomes (Multimedia Appendix 1) such as identifying the bones and muscles of the thoracic wall and summarizing the structure and function of the stomach and small and large intestines, including their location, vascular supply, lymphatic drainage, and nerve supply. They spent about 1 hour at each station before moving on to the next. Each station was managed by a member of the faculty. At the gross anatomy station, students were encouraged to handle the models and specimens to identify key anatomical structures and appreciate their functions and relationships. At the imaging station, the students learned about different imaging modalities and their applications. By watching the real-time cardiac ultrasound, they also learned about the heart’s structure and how it works.

Following the practical session, a TBL (afternoon session of 3 hours) was administered to assess the content and application of the acquired knowledge. Students engaged in the individual readiness assurance test, team readiness assurance test, and application exercise. As part of the TBL process, they actively engaged in the group discussion to share their solutions and rationales. The in-house learning management system was used to administer the TBL activities. These TBL sessions align with the learning outcomes, for example, describe the concept of referred pain and describe the position and functional anatomy of the liver and pancreas (Multimedia Appendix 1). At the end of the practical and TBL, a debrief was given to provide a summary and a take-home message.

A similar teaching and learning plan were followed on day 2 for the gastrointestinal system. However, unlike on day 1, no ultrasound teaching was conducted for the gastrointestinal system; instead, plain and contrast radiographs were used.

#### Survey

At the end of day 2, participants responded to a 4-point Likert scale questionnaire (1: strongly disagree; 4: strongly agree) that collected data about learning resources, learning activities, motivation, and learning experiences. A 4-point scale was chosen, as it would be easily comprehended by younger respondents [27]. Additionally, 3 open-ended questions, that is, aspects of the workshop that were important for student learning, aspects of the workshop that should be changed, and any other additional comments were asked to obtain the free-text responses from students on the usefulness of the outreach session. The questionnaire was distributed via Qualtrics at the end of the program.

### Ethics Approval

Nanyang Technological University institutional review board approval (IRB-2019-09-011) was obtained to use the survey data to evaluate the effectiveness of the outreach program retrospectively. This study involves human participants in the outreach program, albeit with less than minimal risk. The participant information was anonymized for the data analysis. There was no compensation given to the participants for their involvement in this study.

### Data Analysis

Mixed methods were employed to analyze the Likert scale and open-ended responses. The Likert scale scores were reported using descriptive statistics (mean, percentages, and standard deviation). Cronbach α was used to assess the internal consistency in the survey. The data were computed and analyzed using SPSS software (version 26; IBM Corp).

### Thematic Analysis

An inductive thematic analysis was conducted on the free-text comments [28]. First, textual data were stratified according to the answers to the open-ended questions. Two authors (SRM and RV) scanned the text to acquaint themselves with the data before coding. Two researchers then independently analyzed the data to provide initial coding for participant responses. The codes were then compared, and discrepancies were addressed until a resolution was achieved. After consensus, the final themes were described.

## Results

### Participant Demographics

There were 92 initial registrations from 21 different preuniversity academic institutions. Two participants withdrew; so, the final registration number was 90. All 90 participants (16 teams of 5 each) attended the day 1 activity, but only 89 participants attended the day 2 activity. The participant information is summarized in Table 1.

**Table 1:** Demographic information of the participants who were registered for the 2-day outreach (workshop) (N=92).

Qualification of group	Participants, n
Polytechnic year 2	9
Polytechnic year 3	1
International school year Class 12	4
International Baccalaureate year 1	7
International Baccalaureate year 2	17
Junior college year 1	19
Junior college year 2	35

The Ministry of Education schools are the most represented in Singapore with primary and secondary education. The medium of instruction is English. The primary education is compulsory, wherein primary 1 to primary 4 form the foundation stage and primary 5 and primary 6 form the orientation stage. At the end of primary school, the students sit for the Primary School Leaving Examination, and their results will determine the stream that they will enter for secondary education. The secondary education is a 4-year course, leading to Singapore-Cambridge General Certificate of Education O level examination. Following this examination, the students must go through the Joint Admission Exercise, which allows students to apply for admission to courses offered by junior colleges, polytechnic institutes, or the Institute of Technical Education based on their performance.

### Likert Scale Survey Responses

Of the 89 participants who attended the full workshop, 88 participants submitted the survey. Out of the 88 participants, only 81 (92%) fully completed the survey, and their data are included in this analysis. The mean (SD) scores are summarized in Table 2. Most students agreed that the preparatory materials were adequate to guide them through the teaching and learning sessions (mean 3.57, SD 0.57) and that the learning objectives of the course were achieved (mean 3.73, SD 0.52). Regarding faculty, Likert scale assessments revealed that teaching was both clear (mean 3.73, SD 0.52) and effective (mean 3.70, SD 0.53). The participants also provided a positive assessment of the outreach session’s organization (mean 3.64, SD 0.48) and motivation to study medicine or allied/biomedical disciplines (mean 3.69, SD 0.54). Most students also rated their hands-on practical and TBL experiences positively (mean 3.63, SD 0.53 and mean 3.58, SD 0.54, respectively). All participants agreed that they would suggest the course to their peers and juniors. The Cronbach α value of 0.79 indicated good internal consistency in the survey items.

**Table 2:** Mean ratings of the 4-point Likert scale results on the 8 survey items (n=81).

Survey items	Values, mean (SD)
The preparatory materials were sufficient to guide me through the workshop.	3.57 (0.57)
The learning objectives of the workshop were met.	3.73 (0.52)
The professors/tutors at the workshop were clear in teaching the topics.	3.73 (0.52)
The professors/tutors at the workshop were effective in teaching the topics.	3.70 (0.53)
The workshop was well-organized and ran smoothly.	3.64 (0.48)
Attending the workshop has motivated me to pursue medicine or allied health/biomedical sciences.	3.69 (0.54)
How would you rate your learning experience at the hands-on practical session?	3.63 (0.53)
How would you rate your learning experience at the team-based learning session?	3.58 (0.54)

### Themes Derived From the Qualitative Data

Five themes were derived from the qualitative comments as follows.

#### Gaining Perspective

Nearly all the respondents indicated that hands-on experience with real specimens was very important and extremely useful in visualizing the various organs and body systems and how these are connected in real life in a 3D specimen. They believed that this experience was enlightening and provided them with the opportunity to obtain a better picture of the human body structure and function.

…The explaining of anatomy and physiology using plastinated cadavers or models was extremely helpful in helping me to understand and visualize the processes.

…The hands-on practical was very important for me. I could never have dreamt of feeling a heart or a lung before joining medical university.

#### Unique Learning Settings

Most students thought that TBL activities were crucial for their learning and that they could work together, share knowledge, discuss problems, consolidate, and clinically apply the Anatomy and Physiology topics. They thought that the whole experience of working together to learn was more novel, enjoyable, insightful, and captivating than typical lectures.

…Team-based learning was a great experience as I have never done anything like that before and it was really helpful and aided me in communicating well with new peers from other institutions.

…Being able to speak with one another on the cases also enabled us to learn more efficiently as it was more engaging and is a change of pace from the usual lectures.

#### Aspirations and Motivations

According to the students, the outreach program increased their interest and attitude toward medical science and motivated them to seek a career in the medical profession. In addition, they said that this session was beneficial for exposing them to life in a medical school and clarifying prospective medical students’ concerns about applying to medical schools and studying medicine. They valued the interaction with medical students and faculty.

…Very informative and helpful and definitely motivated me to join medicine.

…I think the workshop exposes me to the life of medical school and the first part of MBBS syllabus, which will help me to make informed decision in the future.

#### Sense of Satisfaction

Students reported that the 2-day outreach program had a positive impact on their overall learning experience and attitudes toward medical sciences. They remarked that the learning environment was quite different from that in a typical junior college class, and they would love to come back to learn different body systems. They also suggested carrying on with the session and adding more days to the training program.

…It was an eye-opening experience, and I am glad that LKCMedicine has opened up opportunities for us students to experience this firsthand.

…I would have liked if the workshop were longer (ie, more days for other organs and systems) because I really enjoyed the two days and would have really liked to learn more.

#### Interaction With Facilitators

The participants felt that their understanding of human anatomy and physiology increased because of their interaction with the faculty and medical students, as well as because of the systematic approach, clarity, and precision of the explanations they received.

…The tutors were clear and concise when explaining the various concepts to us.

…the teachers and medical students were a great help in answering our questions.

In addition to the above themes, students suggested improvements, including more days of workshop, providing voice-over PowerPoint presentations, more interactions with students, more hands-on experience with plastinated cadavers, and better time management of the teaching session. Overall, the students’ learning motivation and experiences were positively impacted by the outreach program, as shown by both the Likert scale scores and thematic analyses.

## Discussion

### Principal Findings

The findings from our study strongly support the idea of developing a structured outreach program offering opportunities for preuniversity students to uniquely experience the preclinical environment of learning anatomy and physiology in a medical school. Our study shows that our structured outreach program enhanced students’ interest, motivation, and attitudes toward pursuing medical and allied health courses for their careers.

The learning environment plays a key role in fostering student motivation and learning [24,25]. Extensive comments were made by our study participants on the hands-on learning and visual representations of the topographical arrangements of the anatomical structures and their relationships in human bodies. Our results are comparable to those of prior research that used cadaveric materials in outreach programs [10]. The ability to touch, manipulate, and visualize specimens demonstrates the significance of visual-spatial learning in anatomy because cadavers are typically reserved for use by health science students. Participants in our study had the opportunity to see and touch plastinated human bodies and organs for the first time. Various imaging modalities, virtual dissection, and plastic models were also employed to augment the plastinated materials during the faculty demonstration. This multimodal activity may have stimulated the anatomy practical session and improved students’ understanding of the human body structure [10].

Medical students employ a variety of learning approaches—the commonest being the VARK (Visual, Auditory, Read, and Kinesthetic) model [29]. At LKCMedicine, the teaching method has always been multimodal to cater to students of diverse needs. For example, the TBL material comprises PowerPoint presentations and video lectures (read/auditory/visual of VARK). In the practical class, the plastinated specimens used for teaching are available both in physical form and as a virtual 3D application, accompanied by a demonstration by the facilitators (kinesthetic/visual/auditory of VARK). This implies that individuals with diverse learning styles are afforded the chance to engage with and incorporate various forms of available resources in order to maximize their educational experience. This approach is well supported by evidence [30-33]; therefore, the same experience was provided to the participants of our outreach program.

Outreach activities are extremely effective when they focus on topics that are rarely emphasized in detail in a conventional classroom [34] or clinical applications of biological science principles [6]. This approach is consistent with our study approach, wherein students enjoyed the novel method of learning the anatomy and physiology of the cardiorespiratory and gastrointestinal systems in an integrated and clinically oriented manner through a team-based and hands-on participation. This approach differs from the regular classroom dynamics in that it requires students to communicate with new peers and collaborate to share and develop information. The TBL also involved clinical case vignettes related to common cardiac and gut clinical conditions, which required the application of anatomy and physiology concepts. This method would have provided them with different ways to think about the concept integration and actively discuss the rationale behind their answers within their team and with other teams rather than just memorizing the facts. Several studies have supported the positive benefits and perception of TBL for student learning in higher education [35,36]. In addition, the preuniversity students valued the interaction with faculty and medical students to clarify their questions and improve their understanding. The participants in our study were actively engaged, and they found the program essential for boosting their confidence and assisting them in achieving their long-term medical career objectives. These data show that the student-centric emphasis, clinical application opportunities, and sharing with facilitators contributed to the effectiveness of the outreach session.

Medicine is often regarded as a prestigious and rewarding profession, but junior college students often struggle to connect their coursework to future professions. Like prior research [1,6,7,9,11], our study demonstrates that an anatomy and physiology workshop improves participants’ desire to pursue medicine and other health-related courses. Nonetheless, our outreach session differs from previous research in that our study was observational or flexible in design rather than a realistic model of MBBS classrooms. As in regular class, participants were expected to study pre–reading materials and exposed to multimodal teaching methods during the anatomy practical and TBL activities and have multidisciplinary faculty teach to promote integration of anatomy and physiology with their clinical application. This allows prospective candidates to obtain first-hand knowledge of medical school expectations, pedagogical appropriateness, professors and current medical students, life as a medical student, and the general learning environment. For instance, the flipped class and TBL methods may be preferred by some but not by others owing to differences in teaching and learning styles and preferences [37]. Thus, the outreach activities could emulate the formal undergraduate classes to make informed decisions on the selection of institution and what to pursue for their careers.

Our outreach program differs from previous anatomy or anatomy and physiology programs in terms of its content, length, and delivery style [9,38]. The uniqueness of our outreach program is that it is a replica of the exact learning environment that a first-year medical student would go through in a medical school on a typical day. The transparency of this outreach program allows the students to experience the teaching tools and methods and discuss with faculty, both academic and clinical, who they would potentially interact with if they joined the medical school as part of the TBL and practical.

This outreach was longer than previous anatomy or anatomy and physiology initiatives, but participants in this research advised extending it further. They also recommended adding other body systems to future rounds and were excited to return to the workshop in the future and recommend it to their peers. Despite the outreach being only a 2-day program, participants were highly engaged in the learning process and could conceptualize the basic anatomy and physiology of the cardiorespiratory and gastrointestinal systems in a short span of time. In addition, the participants offered comments and made various suggestions to improve and optimize the course (more engagement with medical students, voice-over PowerPoint presentations, more hands-on practical sessions, time management). This may be another indication of how engaged the participants were and how important they considered the program was.

### Implications

Most educational institutions conduct outreach programs to engage the general community to provide information of their facilities, research, admission processes, available courses, and others. However, this is the first time we have designed and evaluated a unique program bringing in prospective students, providing them a lifelike experience at the medical school and stimulating their decision-making in choosing a career in science and health professions. Although this study explores a novel outreach program in the Asian context, the design of this program has global applications. We suggest other schools adapt some of the aspects discussed in this study and modify the approach toward their outreach goals.

### Limitations of This Study

As the outreach was structured to closely emulate the LKCMedicine’s MBBS preclinical Integrated Anatomy and Physiology session, this study does have limitations. Although the structured session provided better direction to the teaching and team discussions, we did not quantitatively measure the learning outcomes by using preknowledge and postknowledge tests. This is mainly because the session was intended to give preuniversity students an experience of the medical school learning environments and exposure to the anatomy and physiology teaching and learning. Other limitations included participants representing different years of A-levels (equivalent to senior secondary school) and polytechnic courses and limited number of students from each school. Student ability may vary considering their academic, leadership, teamwork, and socioeconomic status.

### Conclusions

The Heart and Guts for Medicine outreach was highly acclaimed by preuniversity students because the program provided them with a near authentic experience as a medical student in the medical school. This study confirms that hands-on learning and collaborative learning in our outreach program increased preuniversity students’ interest in anatomy and physiology and improved their motivation and attitudes toward pursuing medical or allied health courses. These findings might be useful for other educational institutions to plan and organize outreach programs as described in this report. The current standard approach to outreach programs could be modified to transform the open day concept into a comprehensive learning experience. This modification would allow preuniversity students to immerse themselves in the experiences of a medical student.

## Appendix

Multimedia Appendix 1 Learning outcomes of the outreach activity.

## Abbreviations

LKCMedicine: Lee Kong Chian School of Medicine
MBBS: Bachelor of Medicine and Bachelor of Surgery
STEM: science, technology, engineering, and mathematics
TBL: team-based learning
VARK: Visual, Auditory, Read, and Kinesthetic

## Data Availability

All data generated or analyzed during this study are included in this paper and the multimedia appendix.
