# Supplementary material for: Preuniversity Students' Perceptions and Attitudes About an Anatomy and Physiology Outreach Program: Survey Study and Inductive Thematic Analysis
Source: JMIR Form Res. 2024 Aug 12;8:e52533. doi: 10.2196/52533 (PMC11347902; doi:10.2196/52533)
Supplement: Multimedia Appendix 1 [file formative_v8i1e52533_app1.docx]

Learning outcomes – anatomy and physiology outreach program

At the end of this two-day workshop, the workshop participant will be able to:

1. Identify the bones and muscles of the thoracic wall.
2. Describe the topographic anatomy of the lungs and heart.
3. Understand the organisation and functions of different components of the airway.
4. Describe the mechanism of respiration.
5. Recall the nerve supply and blood supply to the airways and heart.
6. Describe the concept of referred pain.
7. Identify the bony and cartilaginous landmarks visible or palpable on the abdominal wall.
8. Recall the descriptive regions of the abdomen including the surface projections of the abdominal organs.
9. Summarise the structure and function of the stomach, small and large intestines including their location, vascular, lymphatic and nerve supply.
10. Describe the position and functional anatomy of the liver and pancreas.
11. Recall the structures associated with the bile secretion and its flow.
